# Supplementary material for: Economy and elderly population, complementary or contradictory: A cross-continental wavelet coherence and cross-country Granger causality study
Source: PLoS One. 2023 Jan 26;18(1):e0278716. doi: 10.1371/journal.pone.0278716 (PMC9879505; doi:10.1371/journal.pone.0278716)
Supplement: S1 Appendix — (DOCX) [file pone.0278716.s001.docx]

**S1 Appendix. Descriptive statistics of the dataset**

| **Countries** | **Descriptive Statistics** | **Variables** | |  |  |
| --- | --- | --- | --- | --- | --- |
|  |  | **GDP (% Annual rate)** | **POP (% of Total Population)** |  |  |
| **All countries** | Obs. | 5,040 | 5,040 |  |  |
|  | Mean | 1.89 | 6.40 |  |  |
|  | Std. Dev. | 4.74 | 4.64 |  |  |
|  | Min. | -47.50 | 1.19 |  |  |
|  | Max | 42.78 | 28.39 |  |  |
| **Africa** | Obs. | 1,620 | 1,620 |  |  |
|  | Mean | 1.13 | 3.81 |  |  |
|  | Std. Dev. | 5.65 | 2.35 |  |  |
|  | Min. | -47.50 | 1.19 |  |  |
|  | Max | 37.53 | 22.55 |  |  |
| **Asia** | Obs. | 900 | 900 |  |  |
|  | Mean | 3.42 | 5.35 |  |  |
|  | Std. Dev. | 4.62 | 3.56 |  |  |
|  | Min. | -29.98 | 2.14 |  |  |
|  | Max | 21.91 | 28.39 |  |  |
| **Europe** | Obs. | 840 | 840 |  |  |
|  | Mean | 2.18 | 14.12 |  |  |
|  | Std. Dev. | 2.98 | 3.76 |  |  |
|  | Min. | -11.23 | 3.47 |  |  |
|  | Max | 13.61 | 23.30 |  |  |
| **North America** | Obs. | 720 | 720 |  |  |
|  | Mean | 1.59 | 5.21 |  |  |
|  | Std. Dev. | 4.31 | 2.57 |  |  |
|  | Min. | -28.64 | 2.46 |  |  |
|  | Max | 23.37 | 20.82 |  |  |
| **Oceania** | Obs. | 180 | 180 |  |  |
|  | Mean | 1.57 | 5.83 |  |  |
|  | Std. Dev. | 4.09 | 4.11 |  |  |
|  | Min. | -19.63 | 2.14 |  |  |
|  | Max | 15.50 | 16.21 |  |  |
| **South America** | Obs. | 780 | 780 |  |  |
|  | Mean | 1.73 | 5.90 |  |  |
|  | Std. Dev. | 4.41 | 2.56 |  |  |
|  | Min. | -15.26 | 2.90 |  |  |
|  | Max | 42.78 | 15.08 |  |  |
