# Supplementary material for: Economy and elderly population, complementary or contradictory: A cross-continental wavelet coherence and cross-country Granger causality study
Source: PLoS One. 2023 Jan 26;18(1):e0278716. doi: 10.1371/journal.pone.0278716 (PMC9879505; doi:10.1371/journal.pone.0278716)
Supplement: S3 Appendix — (DOCX) [file pone.0278716.s003.docx]

| **S3 Appendix: Cross-country analysis from Granger causality test** | | | | | | | | | | |
| --- | --- | --- | --- | --- | --- | --- | --- | --- | --- | --- |
| **Asia** | | | | | **Europe** | | | | | |
| **Country** | **DGDP → DDEPOP** | | **DDEPOP→DGDP** | | **Country** | | **GDP → EPOP** | | **EPOP→GDP** | |
| Myanmar | 0.45 | | 1.23 | | Luxembourg | | 8.6609** | | 1.9029 | |
|  | **GDP → DEPOP** | | **DEPOP → GDP** | |  | | **GDP → DDEPOP** | | **DDEPOP → GDP** | |
| Pakistan | 3.42 | | 6.48* | | Austria | | 6.4806* | | 1.5392 | |
|  | **GDP → DDEPOP** | | **DDEPOP → GDP** | | Belgium | | 4.1877 | | 4.6542 | |
| Bangladesh | 0.12 | | 10.26** | | Denmark | | 8.7784** | | 1.1243 | |
| China | 2.55 | | 10.19** | | Finland | | 1.0453 | | 2.0922 | |
| India | 8.98** | | 0.22 | | France | | 4.7306 | | 3.4062 | |
| Indonesia | 2.67 | | 0.62 | | Greece | | 2.2245 | | 13.109*** | |
| Iran | 0.99 | | 0.87 | | Italy | | 3.1479 | | 2.2305 | |
| Japan | 14.96*** | | 3.14 | | Netherlands | | 0.23701 | | 2.1756 | |
| Korea | 5.73 | | 5.87 | | Norway | | 2.8655 | | 2.5513 | |
| Malaysia | 7.21* | | 0.31 | | Portugal | | 2.0171 | | 4.5149 | |
| Nepal | 8.39** | | 7.38* | | Spain | | 6.558* | | 2.2416 | |
| Philippines | 2.27 | | 0.77 | | Sweden | | 9.8445** | | 4.5765 | |
| Singapore | 8.61** | | 1.02 | | Turkey | | 0.1987 | | 5.5375 | |
| Sri Lanka | 1.84 | | 1.52 | | United Kingdom | | 2.9304 | | 8.7291** | |
| Thailand | 2.34 | | 1.54 | |  | |  | |  | |
|  | | | | | | | | | | |
|  | | | | | | | | | | |
|  | | | | | | | | | | |
|  | | | | | | | | | | |
| **American countries** | | | | | | | | | | |
| **South American Countries** | | **GDP → DEPOP** | | **DEPOP → GDP** | | **North American Countries** | | **GDP → DDEPOP** | | **DDEPOP → GDP** |
| Uruguay | | 2.812 | | 1.159 | | Bahamas | | 3.600 | | 0.743 |
|  | | **GDP → DDEPOP** | | **DDEPOP → GDP** | | Belize | | 0.663 | | 0.413 |
| Argentina | | 0.668 | | 1.461 | | Costa Rica | | 1.815 | | 8.271** |
| Bolivia | | 17.809*** | | 2.145 | | Dominican Republic | | 1.544 | | 0.720 |
| Brazil | | 2.082 | | 0.780 | | Guatemala | | 2.026 | | 0.099 |
| Chile | | 5.867 | | 2.768 | | Haiti | | 0.632 | | 0.922 |
| Colombia | | 6.248* | | 0.132 | | Honduras | | 2.494 | | 9.010** |
| Ecuador | | 0.674 | | 8.353** | | Mexico | | 3.722 | | 4.587 |
| Guyana | | 8.922** | | 0.609 | | Nicaragua | | 1.177 | | 1.934 |
| Latin America & Caribbean | | 1.391 | | 0.842 | | Panama | | 2.169 | | 0.458 |
| Paraguay | | 3.476 | | 0.452 | | Puerto Rico | | 0.461* | | 0.461 |
| Peru | | 8.695** | | 2.247 | | United States | | 1.297 | | 6.099 |
| Suriname | | 1.845 | | 2.566 | |  | |  | |  |
| Trinidad and Tobago | | 0.932 | | 1.823 | |  | |  | |  |

| **Africa** | | | **Oceania** | | |
| --- | --- | --- | --- | --- | --- |
| **Country** | **GDP → EPOP** | **EPOP → GDP** |  | **GDP → DDEPOP** | **DDEPOP → GDP** |
| Mauritania | 0.84228 | 12.601*** | **Australia** | 0.9951 | 1.6186 |
| Niger | 1.3336 | 4.3903 | **Fiji** | 8.4801** | 1.6833 |
|  | **GDP → DEPOP** | **DEPOP → GDP** | **Papua New Guinea** | 1.7055 | 0.6884 |
| Cameroon | 1.8240 | 1.9337 |  |  |  |
| Central African Republic | 14.518*** | 11.577*** |  |  |  |
| Congo, Dem. Rep. | 2.6388 | 2.2607 |  |  |  |
| Nigeria | 4.4071 | 2.3297 |  |  |  |
| Sierra Leone | 3.7257 | 0.9818 |  |  |  |
| Sub - Saharan Africa | 6.967* | 5.3779 |  |  |  |
|  | **GDP →DDEPOP** | **DDEPOP → GDP** |  |  |  |
| Algeria | 1.3726 | 1.8162 |  |  |  |
| Benin | 6.3329* | 0.0872 |  |  |  |
| Botswana | 14.774*** | 0.6548 |  |  |  |
| Burkina Faso | 0.8846 | 1.8128 |  |  |  |
| Burundi | 6.391* | 3.3639 |  |  |  |
| Congo, Rep. | 0.27751 | 0.39778 |  |  |  |
| Cote d'Ivoire | 0.31018 | 0.5676 |  |  |  |
| Egypt, Arab Rep. | 0.57814 | 5.283 |  |  |  |
| Gabon | 4.5213 | 16.104*** |  |  |  |
| Ghana | 3.8879 | 2.5305 |  |  |  |
| Kenya | 1.8728 | 0.68504 |  |  |  |
| Lesotho | 5.0136 | 2.6629 |  |  |  |
| Madagascar | 7.7318* | 3.0313 |  |  |  |
| Malawi | 8.3236** | 0.2221 |  |  |  |
| Rwanda | 1.0343 | 5.4212 |  |  |  |
| Senegal | 6.9288* | 8.7281** |  |  |  |
| Seychelles | 1.2034 | 2.3959 |  |  |  |
| Sudan | 2.3360 | 1.4576 |  |  |  |
