# Supplementary material for: Economy and elderly population, complementary or contradictory: A cross-continental wavelet coherence and cross-country Granger causality study
Source: PLoS One. 2023 Jan 26;18(1):e0278716. doi: 10.1371/journal.pone.0278716 (PMC9879505; doi:10.1371/journal.pone.0278716)
Supplement: S4 Appendix — (DOCX) [file pone.0278716.s004.docx]

**Appendix S4: Impulse response of the per capita GDP and elderly population for all countries & Root of the companion matrix**

Appendix S4.1: Impulse response of the per capita GDP and elderly population for all countries

Source: Authors’ illustration based on data.

**Appendix S4.2: Roots of the companion matrix**

Source: Authors’ illustration based on data.
